# Case of Encysted Sanguineous Tumor of the Neck, Successfully Removed

**Published:** 1856-05

**Authors:** J. M. Carnochan

**Affiliations:** Surgeon-in-Chief to the State Hospital, Professor of Surgery in the New York Medical College, etc.


*A Case of Encysted Sanguineous Tumor of the Neck, successfully removed.* By J. M. Carnochan, M. D., Surgeon-in-Chief to the State Hospital, Professor of Surgery in the New York Medical College, etc.

[From the American Medical Gazette, February, 1856.]

I give the name of encysted sanguineous tumor to a tumor met with on the lateral aspect of the neck; at times projecting in front of the anterior margin of the sterno-mastoid muscle, but sometimes found more prominent behind the posterior margin of that muscle. The essential anatomical characters of this tumor are a cyst of greater or less extent and thickness, containing fluid and coagulated blood with fibrin, and a rough and irregular internal surface, bearing much resemblance to the inner surface of an aneurismatic sac; while the external surface has also the appearance presented externally by an aneurism, when exposed by dissection. This kind of tumor is connected with the sheath of the large vessels of the neck, and is seated under the sterno-mastoid muscle and deep cervical fascia. As far as observation and inquiry inform me, it is more common in females than in males—in fact, the only cases of which I am cognizant, were met with in adult females. If the cyst is opened at once by a free incision, the hemorrhage is great and alarming; blood pouring out profusely from the interior of the sac, and gushing like water through sand, at the bottom of a well. If, however, the cyst be opened by a small incision, gradually enlarged, it will collapse slowly upon its decreasing contents, and the hemorrhage will be comparatively trivial and easily controled.

Various kinds of encysted tumors of the neck have been noticed by surgical writers, but I am not aware that there is any account extant of a tumor presenting the same characters as the one I have just described. Maunoir, of Geneva, gives a description of a tumor of the encysted form, to which he gives the name of "hydrocele of the neck;" the contents of the cyst being generally limpid, but presenting also, at times, a reddish, chocolate, or greenish color, with micaceous particles floating on the surface. Such tumors are, for the most part, seated on the left lateral aspect of the neck; they are small when first perceived, but increase so as to occupy a great portion of the side of the neck, and may become so large as to interfere with respiration. Encysted tumors of the neck are also met with as congenital. In such instances, the general enlargement is found to be composed of groups, or of a large number of smaller cysts. According to Mr. Hawkins, of London, such cysts contain a transparent fluid; although, at times, the contents have a reddish tint, or are dark colored, like venous blood, but without coagulum, being evidently a colored secretion. These various forms of encysted growths are distinct from tumors of the Thyroid Gland, and entirely different from the tumor described by Boyer, which is formed by a development of the *bursa* found between the thyroid cartilage and the *os hyoides*.

Three cases of "encysted sanguineous tumor" have come under my notice; all of them having the same characters. One of these occurred several years ago in the practice of my illustrious friend, Professor Mott, and had to be abandoned, on account of the enormous hemorrhage, which immediately followed the opening of the cyst, during the operation; another occurred in the practice of an eminent surgeon of this city, and was also abandoned during the dissection, on account of the similarity of the cyst to an aneurism; and the third is the case related below, and which, although the source of solicitude to me during the operation, I succeeded in removing, with a favorable result.

In the beginning of September last, I was consulted by Mrs. R. S., a lady from the State of Vermont, aged 36 years, of nervous temperament, and apparently in feeble health. About twelve years ago, she perceived a small enlargement about half an inch below the lobe of the ear, on the right side, and on a line with the large vessels of the neck. For a period of eight years the progress of the tumor was very gradual, attracting but little attention, and causing only trifling inconvenience. Within the last four years it had continued to increase more perceptibly, and during the last year the enlargement had progressed so rapidly as to prove the source of much anxiety, as well as of pain and inconvenience, especially during respiration. When shown to me for my advice, the tumor was as large as a goose egg, extending downwards, from a line on a level with the lobe of the ear, to within an inch of the upper border of the clavicle, and occupying the right side of the neck, before and behind the margin of the sterno-mastoid muscle, under which it was placed.

The tumor passed forwards auteriorly, so as to encroach upon the lobe of the thyroid body, while posteriorly it occupied partly the space lying between the sterno-mastoid aud trapezius muscles. The tumor was slightly oblong, smooth, immovable at the base and tense, as if bound down by the deep cervical fascia and the sterno-mastoid muscle. Upon manual examination, a sensation of fluctuation was imparted, similar to that which characterizes encephaloid tumors.

The pulsations of the carotid artery were transmitted to it, giving it in this respect an aneurismatic feature. The binding down, also, of the tumor by the superimposed tissues, gave an indefinable character to the outline of the growth. The diagnosis was consequently obscure, and, upon the first examination, I was not certain that the disease was not one of malignant character.

The patient was exceedingly anxious to be freed from her malady, and I assented to perform the operation, as soon as she recovered from the fatigue of her journey to the city. The diagnostic inference I had drawn from the examination of the tumor was, that the growth was one of the encysted kind, with a dense and thickened sac, or that it was encephaloid in its character, or a gland which had assumed the structure mentioned by Abernethy as the *vascular sarcoma*. With these views, I undertook the operation, at the patient's residence, on the 7th of September last, in the presence of my colleagues, Professors Horace Green and E. H. Parker, and several other medical gentlemen, and aided by my friends, Drs. Proudfoot, J. Crane, Casseday and E. Morehead.

*Operation.*—The operation was literally a careful dissection of the lateral portion of the neck, complicated by a morbid growth in the midst of the most important structures.

The patient was placed upon a table of suitable height, and after the exhibition of chloroform, the shoulders were elevated on a pillow, and the face turned towards the unaffected side. The assistants being properly placed, an incision was made, commencing half an inch below the lobe of the ear, extending nearly vertically downwards over the middle of the tumor, and terminating an inch and a half above the clavicle. The integuments, platisma myoides, and superficial fascia were thus divided, the external jugular vein being cautiously avoided. The dissection was now proceeded with, so as to expose the fibres of the sterno-mastoid muscle and the deep cervical fascia, where the latter covered that portion of the tumor which projected behind the posterior margin of the sterno-mastoid, in the upper part of the supra-clavicular triangle. The deep attachments of the tumor became now still more evident, as well as the firm manner in which it was bound down by the sterno-mastoid and the deep fascia of the neck. A director was now carefully insinuated between the sterno-mastoid muscle and the deep cervicae fascia, and the fibres of the muscle were divided transversely, at a point where the muscle lay over the centre of the tumor. The tumor was next laid bare, still invested by the layer of the deep cervical fascia. At this stage of the dissection, the bulk of the tumor seemed to lie over the tract of the large vessels, and the pulsations imparted to it by the beating of the carotid artery, induced a general belief that the tumor was an aneurism of that vessel. With the forceps, a *dents de souris*, the deep fascia was pinched up at the most prominent part of the tumor, and a small aperture made in it. A director was then carefully insinuated between the investing fascia and the tumor, and the fascia slit to the extent of an inch and a half. The surface of the tumor was now entirely denuded at that part, and the aneurismal similarity became still more apparent. To the pulsations were added, to complicate the diagnosis, the sensation of fluctuation and the dark red color appertaining to the external surface of an aneurismal sac. Some of the lookers-on became appalled; the destruction of the patient on the table, from the bursting of the supposed aneurism, seemed inevitable; but my own opinion was that, under the circumstances, the best practice would not be to close the wound and leave the patient to her fate. Relying on the diagnosis I had formed, I could not believe that the tumor was an aneurism, and the case already alluded to above, at which I had assisted Professor Mott some years ago, coming to my mind, I judged that I had encountered an encysted sanguineous tumor.

Deciding to proceed with the dissection, I prepared for contingencies that might arise, and more especially recollecting also the frightful hemorrhage in Professor Mott's case, I resolved to avoid, if possible, an abrupt or sudden incision into the cavity of the tumor, and to allow the influence of atmospheric pressure to take effect gradually upon the bleeding vessels, upon the inner surface of the sac. And further, if the tumor were to prove an aneurism of the carotid, I should tie the common trunk below and above the tumor; if it turned out to be an encephaloid growth, I should carry the dissection as far as necessary, without inflicting a mortal wound; and if it happened to be an encysted sanguineous tumor, the best plan was to remove it.

As a preliminary step, the flow of blood from the surface was entirely arrested, and the wound sponged and cleansed with ice water. As the trunk of the carotid below the tumor was within easy reach, I proposed now to make a small puncture into the tumor, and put its real character to a definite test. A sharp-pointed grooved director was accordingly carefully inserted into the tumor, and blood immediately flowed along the groove in considerable quantity. This was still apparently confirmatory that the tumor was aneurismatic. Withdrawing the pointed director, I passed a narrow blunt-pointed tenotome into the puncture just made, and enlarged the opening upwards about a quarter of an inch. The hemorrhage from this aperture was considerable, but not profuse. It was now evident that a sac was opened, and that it was filled with blood and coagulum. By pressure, applied upon the sides of the sac, about a tablespoonful of coagulated blood was squeezed out, and fluid blood continued to flow, but in diminished quantity. The sac, by continuing the pressure, began to collapse, and to be filled more slowly, when a portion of the contents were evacuated. Taking the tenotome again, the aperture was gradually enlarged, by prolonging the incision upwards for a quarter of an inch more. About an ounce more of coagulum was squeezed out, and the fluid blood was supplied more slowly. The incision of the sac was again slowly enlarged for half an inch more; but little blood flowed, and the interior of the sac could be seen partly filled up with coagulum and fibrin. It was now very evident that the sac could not communicate with any large arterial trunk, and that the tumor could not be other than an encysted unilocular bloody tumor. It now remained to detach the tumor from its extensive connections, which still remained. The tumor, taking its origin within the sheath of the vessels, by gentle touches with the scalpel and the use of the handle of the instrument, was carefully detached from the carotid, anteriorly and inferiorly. The *par vagum* now came into view posteriorly, and where the tumor extended towards the supra-clavicular triangle, its deep surface was detached in the same manner from the internal jugular vein. At the upper part of the tumor, the sac dipped between the common carotid and the jugular vein, separating these vessels, and extending deeply as far as the styloid process and the transverse processes of the adjoining cervical vertebræ. This upper horn, as it were, of the cyst, surrounded by so many important parts, was carefully detached, until the tumor remained attached only by a small pedicle, firmly adherent to the transverse process of the third cervical vertebra. A strong ligature was carried to the bottom of the wound, around the highest part of the pedicle, and tied firmly. A few cautious touches with the knife divided the pedicle, the last attachment of the tumor to its connections. About eight ligatures being applied to some branches which were divided during the operation, the bleeding entirely ceased, and the patient was allowed to come out of the anæsthetic influence, under which she had been for half an hour, the time occupied in the performance of the operation.

The wound was brought together by sutures and adhesive straps. Slight suppuration took place at the upper part of the incision, where remained the ligature around the pedicle. This ligature came away on the tenth day. No untoward complication retarded the progress of cure, and twenty-five days after the operation Mrs. S. left the city for her home, the wound entirely cicatrized, and in other respects in good health.

The sac of the tumor, even after its removal, resembled in a remarkable degree a true aneurismatic sac. The external surface was smooth and of a dark red color; the internal was rough, irregular, and lined with fibrin; while the contents of the sac were composed of fibrinous material, fluid, and coagulated blood. The thickness of the wall of the tumor was about equal to that of an aneurism of the aorta.

NO. 2 WAVERLY PLACE, JAN. 15, 1856.

